# Predictors of Unfavorable Therapeutic Response to Left Bundle Branch Area Pacing and Atrioventricular Node Ablation in Patients with Atrial Fibrillation and Heart Failure

**DOI:** 10.3390/jcdd13070330

**Published:** 2026-07-14

**Authors:** Lishui Shen, Hailing Li, Wenliang Che, Kai Tang, Dongdong Zhao

**Affiliations:** Department of Cardiology, Shanghai Tenth People’s Hospital, Tongji University, Shanghai 200072, China; shenlishui@163.com (L.S.);

**Keywords:** atrial fibrillation, heart failure, left bundle branch area pacing, atrioventricular node ablation

## Abstract

Background: Left bundle branch area pacing (LBBAP) with atrioventricular node (AVN) ablation has emerged as a promising therapy for patients with atrial fibrillation (AF) and symptomatic heart failure (HF). However, it remains unclear which patients are likely responders and which may derive limited benefit. Objective: This study aimed to identify predictors of unfavorable therapeutic outcomes following this ‘ablate and pace’ therapy. Methods: In this retrospective analysis, consecutive patients with successful LBBAP and AVN ablation were included. Baseline characteristics and long-term outcomes were assessed. Multivariable Cox regression and reclassification analysis were used to evaluate the predictors of heart failure hospitalization (HFH). Results: Ninety-eight patients (age 73.1 ± 8.9 years; ventricular rate 93.6 ± 18.7 bpm; 41.8% male) were enrolled. During 43.9 ± 19.4 months of follow-up, 19 (19.4%) patients experienced HFH. Three independent risk factors were identified for HFH: impaired renal function (eGFR ≤ 60 mL/min/1.73 m^2^; HR: 7.9), left atrial enlargement (LAD ≥ 50 mm; HR: 4.8), and male sex (HR: 3.9). A risk stratification model incorporating these factors effectively discriminated patients with minimal versus high HFH risk. All patients with three risk factors experienced HFH within 2 years, whereas none of the 25 patients without any risk factors experienced the endpoint during 113 patient years of follow-up. Conclusions: The eGFR ≤ 60 mL/min/1.73 m^2^, LAD ≥ 50 mm, and male sex were likely predictors of poor therapeutic outcomes to ‘ablate and pace’ therapy in patients with AF and HF. Patients without any of these risk factors had minimal HFH risk, whereas ≥2 risk factors showed a markedly higher risk. This may help to identify potential patients who are less likely to benefit from this combined approach, although prospective validation is needed before clinical application.

## 1. Introduction

Permanent pacemaker implantation combined with atrioventricular node (AVN) ablation serves as an effective therapeutic strategy for controlling and regularizing the ventricular rate in patients with atrial fibrillation (AF) and symptomatic heart failure (HF) [1]. Previous meta-analysis revealed that ‘ablate and pace’ therapy could improve a broad range of clinical outcomes, including ventricular function, quality of life, exercise duration, and healthcare use [2]. However, conventional right ventricular pacing (RVP) may result in left ventricular dyssynchrony, potentially diminishing the benefits of the rate control strategy [3,4,5]. Left bundle branch area pacing (LBBAP), a more physiological conduction system pacing modality, has emerged as an alternative to mitigate the adverse effects associated with RVP [6,7]. Growing evidence indicates that in patients with AF and HF, LBBAP following AVN ablation offers comparable cardiac function benefits to those of biventricular pacing [8,9].

However, in clinical practice, a subset of patients remains at risk for HF episodes despite this treatment. In the study by Cai et al., 10.1% of patients experienced heart failure hospitalization (HFH) after ‘ablate and pace’ therapy, while in the study by Vijayaraman et al., this proportion was as high as 39% [10,11]. Currently, it remains unclear which patients are likely responders and which may derive limited benefit. This study aimed to identify risk factors predictive of poor therapeutic outcomes.

## 2. Methods

### 2.1. Study Population

This study was performed at the Shanghai Tenth People’s Hospital between March 2019 and December 2021. All consecutive patients who met the following inclusion criteria were recruited: (1) refractory persistent AF with symptomatic HF despite optimal medical therapy (i.e., refractory to pharmacological rate or rhythm control, unsuitable for catheter ablation or in cases where ablation failed), and (2) received successful AVN ablation and LBBAP. We excluded patients who were aged <18 years, had stage 5 CKD, or had a life expectancy of <1 year. Written informed consent was obtained from all participants. This study complied with the principles of the Declaration of Helsinki and was approved by the institutional ethics committee of Shanghai Tenth People’s Hospital.

### 2.2. Left Bundle Branch Area Pacing

The procedural steps for delivering LBBAP have been described previously [12,13]. A 4.1 Fr bipolar active fixation lead (Select Secure 3830, Medtronic, Minneapolis, MN, USA) was delivered through a pre-shaped long sheath (C315-His, Medtronic, Minneapolis, MN, USA). The His bundle was located by recording the His bundle potential in the Medtronic 3830 lead. Afterwards, the LBBAP lead was positioned approximately 1 cm distal to the His bundle position in the RV septum. The eligible site for LBBAP showed a negative “W” waveform on lead V1 during activation mapping. The electrode was then manipulated perpendicularly to the interventricular septum and screwed clockwise to reach the left ventricular subendocardium. The criteria for successful LBBAP included the fluoroscopic fulcrum sign, paced morphology of the right bundle branch block (RBBB) pattern and the stimulus-peak left ventricular activation time shortening abruptly with increasing output or remaining short and constant at low and high outputs. The intrinsic and paced QRS duration and stimulus-peak left ventricular activation time were measured and optimized to mimic physiological conduction. A right ventricular (RV) backup lead was implanted at the operator’s discretion.

### 2.3. Atrioventricular Node Ablation

AVN ablation was performed at the time of pacemaker implantation or at a later date, depending on the physician’s discretion and patient’s clinical circumstances. An 8.5 Fr long sheath (SL1; Fast-Cath, Abbott, Abbott Park, IL, USA) was inserted through the femoral vein to access the AV junction region, encompassing the AVN and proximal His bundle area. An 8 Fr 3.5 mm tip ablation catheter (ThermoCool^®^ SmartTouch, Biosense Webster, CA, USA) was advanced through the sheath to perform the AVN ablation. We used the right anterior oblique (RAO) view for positioning of the catheter tip to the presumed area of the AVN. The location was optimized according to the intracardiac electrograms. Radiofrequency ablation was performed until AV block was established. In cases with failure to achieve AV block from the right side, the ablation catheter was advanced through the femoral arterial access to ablate the His bundle potential site in the left ventricle. All patients were monitored for 20 min post-ablation to ensure no return of AV conduction after achieving AV block. Following AVN ablation, the lower pacing rate was initially set at 90 bpm and decreased to 70 bpm at 1 to 3 months after the procedure. One case example of LBBAP and AVN ablation is shown in Figure S1.

### 2.4. Data Collection and Clinical Follow-Up

Baseline characteristics including laboratory evaluation, echocardiographic parameters, New York Heart Association (NYHA) functional class, and medications were collected. Device interrogation was routinely performed at 1, 3, 6 months and then annually after implantation. Echocardiography was examined every 3 to 6 months and at the time of symptoms. The primary outcome was HFH, defined as the first episode of unplanned hospitalization lasting more than 24 h due to signs and symptoms consistent with congestive HF and requiring intravenous therapy. The secondary outcome was all-cause death and thromboembolic event. The thromboembolic event included ischemic stroke, transient ischemic attack, and peripheral vascular thrombosis. In this study, unfavorable therapeutic response was defined as the occurrence of HFH during follow-up.

### 2.5. Statistical Analysis

Continuous data are summarized as mean ± SD or median (interquartile range). Categorical variables are expressed as absolute frequencies and percentages. Comparison between groups was performed with the t-test or Mann–Whitney U test for continuous variables and the Chi-square test or Fisher’s exact test for categorical variables. Changes in echocardiographic parameters and NYHA classification from baseline to the 12-month follow-up were analyzed using paired t-tests. The impact of various clinical variables on HFH-free survival was explored using the Cox proportional hazards model. Variables associated with HFH were initially identified through univariate analysis; those with a *p*-value < 0.10 underwent further multivariate analysis. The added predictive value of incorporating new parameters into the models was evaluated through reclassification analysis, including integrated discrimination improvement (IDI) and continuous net reclassification improvement (NRI). Model discrimination was further compared using C-statistics, and the goodness of fit was assessed via the Akaike information criterion (AIC). A two-tailed *p*-value < 0.05 was considered statistically significant. Statistical analyses were performed using SPSS 22.0 software (SPSS Inc., Chicago, IL, USA) and Stata 17 (StataCorp LLC, College Station, TX, USA).

## 3. Results

### 3.1. Patient Characteristics

Ninety-eight patients were enrolled in this study. The baseline characteristics of the study population are shown in Table 1. The mean age was 73.1 ± 8.9 years, and 41.8% of the patients were male sex. Baseline ventricular rate was 93.6 ± 18.7 bpm. The average eGFR was 68.6 ± 21.7 (mL/min/1.73 m^2^), and 39.8% of patients had an eGFR ≤ 60 mL/min/1.73 m^2^. The CHA2DS2-VASc and HAS-BLED scores were 4.9 ± 1.8 and 2.5 ± 1.1, respectively. The average LAD was 47.8 ± 7.2 mm, and 30.6% of patients had an LAD ≥ 50 mm. Baseline LVEF was 54.5 ± 8.0%, and 64.3% of patients were in NYHA class III or IV.

CHA_2_DS_2_-VASc is a score to evaluate embolic risk for atrial fibrillation population incorporating congestive heart failure, hypertension, age (two scores for ≥75 y, and one score for age 65–74 y), diabetes mellitus, stroke/transient ischemic attack/thromboembolism history, vascular disease, female sex. HAS-BLED a score to evaluate major bleeding risk for the atrial fibrillation population incorporating hypertension, abnormal liver/kidney function, stroke history, bleeding history, labile international normalized ratio, elder age, and drug predisposing bleeding/alcohol abuse.

ACEi = angiotensin-converting enzyme inhibitor; ARB = angiotensin receptor blocker; ARNI = angiotensin receptor neprilysin inhibitor; AVN = atrioventricular node; eGFR = estimated glomerular filtration rate (calculated by CKD-EPI formula); HFH = hospitalization for heart failure; HFmrEF = heart failure with mildly reduced ejection fraction; HFpEF = heart failure with preserved ejection fraction; HFrEF = heart failure with reduced ejection fraction; LAD = left atrial anterior–posterior diameter; LBBB = left bundle branch block; LBBAP = left bundle branch area pacing; LVEDd = left ventricular end-diastolic diameter; LVEF = left ventricular ejection fraction; MRA = mineralocorticoid receptor antagonist; NT-proBNP = N-terminal-pro hormone B-type natriuretic peptide; NYHA = New York Heart Association; RA = right atrium; RBBB = right bundle branch block.

### 3.2. Periprocedural Outcomes

All device implantations and AVN ablations were acutely successful. Thirteen (13.3%) patients implanted a single-chamber pacemaker and 85 (86.7%) patients implanted a dual-chamber pacemaker (Table 2). The intrinsic and paced QRS durations were 97.5 ± 18.4 ms and 126.1 ± 10.5 ms, respectively. LBBAP with a capture threshold <1.0 V at 0.4 ms was achieved in 95 (96.9%) patients. During bipolar pacing testing, all patients achieved anodal capture, with complete elimination of the RBBB pattern in 68 (69.4%) patients. The mean sensed R waves were 12.1 ± 3.8 mV in bipolar tip-ring configuration.

Overall, 99 AVN ablation procedures were performed. Simultaneous pacemaker implantation and AVN ablation were performed in 89 (90.8%) patients. Nine (9.2%) patients underwent AVN ablation one month after pacemaker implantation, with an average interval of 1.6 ± 0.5 months (range 1.1–2.6 months). One patient with successful initial procedure who later regained AV nodal conduction required a repeat AVN ablation procedure. No patient observed an acute increase in capture threshold after AVN ablation. There was no significant change in paced QRS duration before and after AVN ablation (126.1 ± 10.5 ms vs. 126.0 ± 10.0 ms, *p* = 0.902).

Echocardiographic Parameters, NYHA Functional Class and Pacing Parameters at 1-year Follow-up.

**Figure 1:**
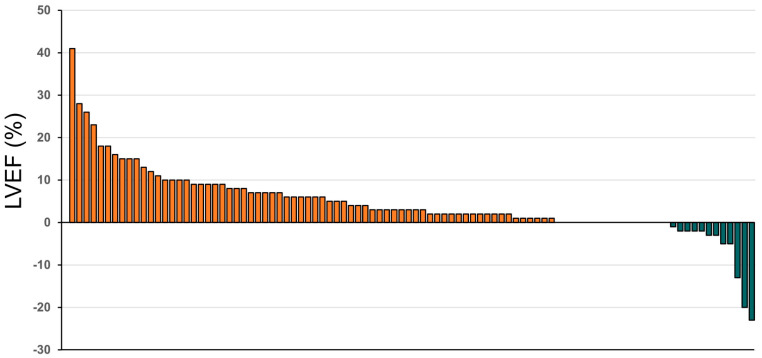
Distribution of changes in left ventricular ejection fraction in each individual at 1-year follow-up.

**Figure 2:**
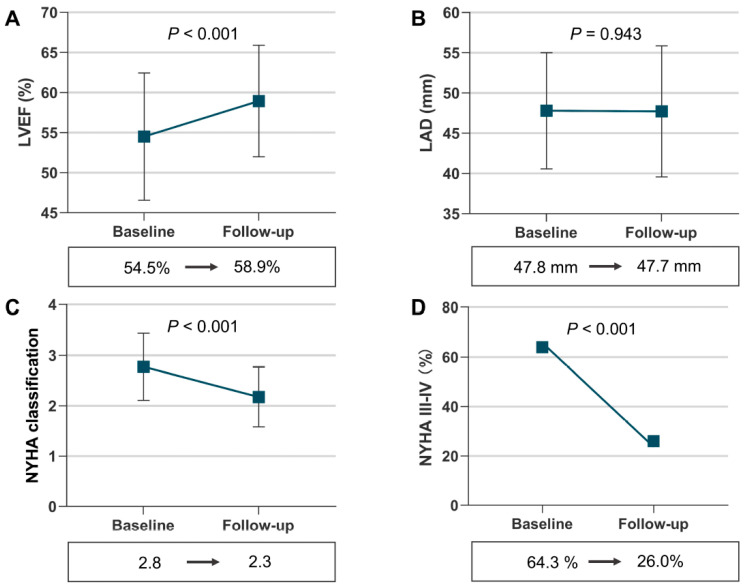
Comparison of changes in echocardiographic parameters and New York Heart Association (NYHA) functional class between baseline and 1-year follow-up after left bundle branch area pacing plus atrioventricular node ablation. (**A**) Mean left ventricular ejection fraction (LVEF) increased from 54.5% to 58.9%. (**B**) Mean left atrial anterior–posterior diameter (LAD) showed no significant change. (**C**) Mean NYHA classification improved from 2.8 to 2.3. (**D**) Proportion of NYHA functional class III-IV decreased from 64.3% to 26.0%.

Ninety-six patients (98.0%) completed a 1-year follow-up. Two patients died of heart failure within 1 year. Figure 1 shows the rate of change in LVEF observed in all study participants. As presented, an absolute increase in LVEF was observed in 68 of 96 (70.8%) patients. Twelve (12.5%) patients showed an absolute decrease in LVEF. The mean LVEF increased from 54.5% ± 8.0% at baseline to 58.9% ± 7.0% at 1 year, the mean NYHA functional classification improved from 2.8 ± 0.6 to 2.3 ± 0.8, and the proportion of NYHA functional class III to IV decreased from 64.3% to 26.0% (all *p* < 0.001, Figure 2). Baseline LVEF was reduced (<50%) in 18 patients (18.4%). In the reduced LVEF group, the mean LVEF was increased from 41.3% ± 8.7% to 53.3% ± 9.6% (*p* < 0.001). In the preserved LVEF group, the mean LVEF was increased from 57.5% ± 3.5% to 60.2% ± 5.6% (*p* < 0.001).

**Figure 3:**
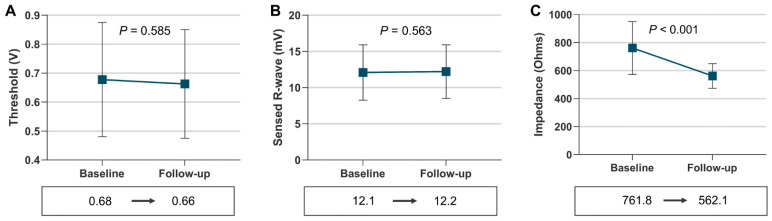
Electrical parameters of left bundle branch area pacing at implant and at 1-year follow-up. (**A**) Pacing threshold. (**B**) Sensed R-wave amplitude. (**C**) Lead impedance.

The LBBAP parameters are summarized in Figure 3. There was no significant change in capture threshold and bipolar sensed R wave at 1-year follow-up (all *p* > 0.05). No patient exhibited an increase in the threshold above 2.0 V at 0.4 ms. The mean LBBAP lead impedance was found to decrease at 1-year follow-up (761.8 ± 188.5 vs. 562.1 ± 88.1 Ohms, *p* < 0.001).

### 3.3. Long-Term Clinical Outcomes

Over a mean follow-up of 43.9 ± 19.4 months, 19 (19.4%) patients occurred HFH. The median time from the procedure to the HFH event was 5.0 [range, 1.7–28.9] months. HFH patients showed no significant improvements in LVEF (53.7 ± 8.8% vs. 52.6 ± 9.2%, *p* = 0.432) and NYHA functional classification (2.9 ± 0.8 vs. 2.9 ± 1.0, *p* = 0.826) at 1-year follow-up. Moreover, HFH patients showed a higher proportion of male sex (63.2% vs. 36.7%, *p* = 0.036), more reduced renal function (eGFR, 50.6 ± 18.1 vs. 73.0 ± 20.3 mL/min/1.73 m^2^, *p* < 0.001), and larger LAD (52.9 ± 9.5 vs. 46.6 ± 6.0 mm, *p* < 0.001) (Table 1). There was no difference in pacing and ablation characteristics between patients with and without HFH (Table 2).

Five patients died during the follow-up period. The cause of death was decompensated heart failure in two patients; two patients died of severe COVID-19 pneumonia and multiple organ failure, and one cause of death was lung cancer. Four patients (4.1%) occurred thromboembolic events. No hemorrhagic stroke or gastrointestinal hemorrhage was reported.

### 3.4. Risk-Prediction Model

**Figure 4:**
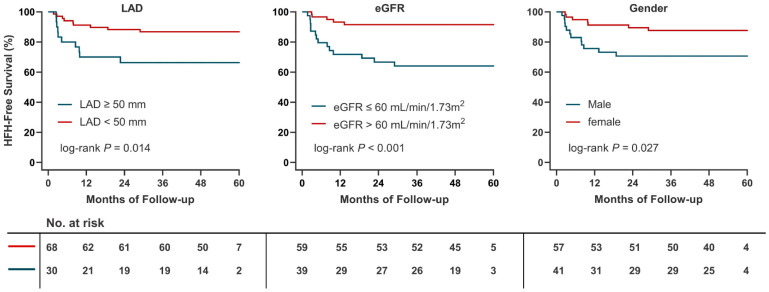
Survival free from heart failure hospitalization. Survival curves of 98 patients undergoing left bundle branch area pacing plus atrioventricular node ablation categorized by predictors of heart failure hospitalization. Patients with eGFR ≤ 60 mL/min/1.73 m^2^, LAD ≥ 50 mm, and male sex had worse therapeutic efficacy than patients not fulfilling these criteria. eGFR = estimated glomerular filtration rate (calculated by CKD-EPI formula); LAD = left atrial anterior–posterior diameter.

**Figure 5:**
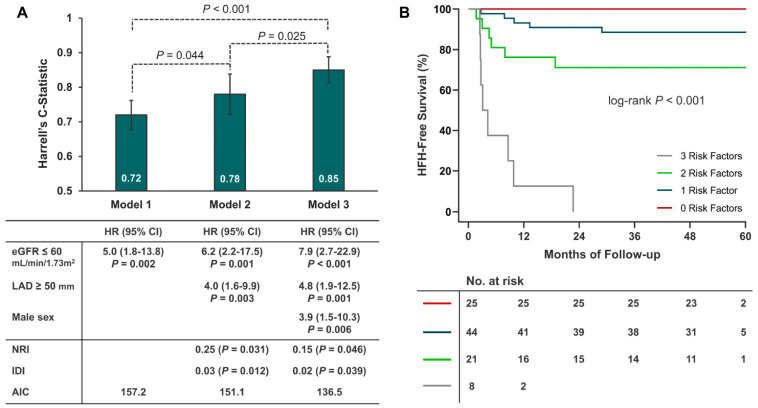
Prediction models. Incremental value of combining risk markers to predict heart failure hospitalization in 98 patients undergoing left bundle branch area pacing plus atrioventricular node ablation (**A**), and Kaplan–Meier curves of survival free from heart failure hospitalization (**B**). AIC = Akaike information criterion; CI = confidence interval; eGFR = estimated glomerular filtration rate (calculated by CKD-EPI formula); HR = hazard ratio; IDI = integrated diagnostic improvement; LAD = left atrial anterior–posterior diameter; NRI = net reclassification improvement; Model 1 = only eGFR; Model 2 = eGFR and LAD; Model 3 = eGFR, LAD, and gender.

Cox proportional hazards regression analysis revealed that eGFR ≤ 60 mL/min/1.73 m^2^ (HR: 7.9, 95% CI: 2.7–22.9, *p* < 0.001), LAD ≥ 50 mm (HR: 4.8, 95% CI: 1.9–12.5, *p* = 0.001) and male sex (HR: 3.9, 95% CI: 1.5–10.3, *p* = 0.006) could independently predict the risk of HFH (Table 3, Figure 4). Adding LAD ≥ 50 mm to eGFR ≤ 60 mL/min/1.73 m^2^ significantly increased Harrell’s C-statistic and improved risk reclassification. Adding male sex to the combination of eGFR ≤ 60 mL/min/1.73 m^2^ and LAD ≥ 50 mm further improved C-statistic significantly and improved risk reclassification (Figure 5A). All patients with three risk factors experienced HFH within 2 years, whereas none of 25 patients without any risk factors experienced the endpoint during 113 patient years of follow-up. There was no significant increase in risk from no risk factors to one risk factor, but a five-fold increase from one to two risk factors (HR: 4.7, 95% CI: 1.8–30.3, *p* = 0.034) and almost a 17-fold increase in risk from two to three risk factors (HR: 16.8, 95% CI: 4.2–68.2, *p* < 0.001) (Figure 5B).

## 4. Discussion

The main findings of this single-center, retrospective study are as follows: (1) AVN ablation and LBBAP can improve LVEF and NYHA functional class in the majority of patients with refractory AF and HF; (2) however, approximately 20% of patients fail to derive cardiac function benefits from this combined therapy and still experience HFH events; (3) eGFR ≤ 60 mL/min/1.73 m^2^, LAD ≥ 50 mm, and male sex are predictors of poor therapeutic outcomes, and the combination of these improved the prediction of HFH risk significantly; (4) patients with all three risk factors had almost 17-fold risk compared with those who had two risk factors, indicating that the combined therapy of AVN ablation and LBBAP may derive limited benefits in this particular population.

As a promising modality of physiological pacing, LBBAP addresses dyssynchrony issues often seen with right ventricular pacing, potentially offering better protection of heart function. This pacing strategy, increasingly utilized to manage rate control in AF patients undergoing AVN ablation, has shown promise in improving life quality and clinical outcomes [14,15]. A recent study by Wang et al. reported that LBBAP combined with AVN ablation can significantly reduce the incidence of inappropriate shocks and improve LV function in persistent atrial fibrillation patients with heart failure and ICD implantation [16]. Cai and colleagues demonstrated that this therapy can increase LVEF in patients with either reduced or preserved LV systolic function [10]. Similarly, our study also observed improvements in NYHA functional class and LVEF following this combined therapy. By regularizing rhythm and heart rate and maintaining a physiological contraction through physiologic pacing, LBBAP plus AVN ablation could reduce cardiac workload, and favor the restoration of normal intracardiac pressures and volumes; there will also be a reduction in atrial and ventricular stretch [17]. This would promote a cardiac reverse remodeling mechanism, responsible for improving heart function [18].

Nevertheless, it is noteworthy that not all patients derive benefits from this intervention. Previous reports have indicated that 1.5% to 39% of patients undergoing CSP plus AVN ablation remained at risk of HFH [8,19,20,21,22,23]. In the present study, 19.4% of patients experienced HFH events within 5 months after ‘ablate and pace’ therapy. Moreover, these patients demonstrated no improvement in LVEF or NYHA functional classification, and two cases occurred heart failure-related death. Indeed, AF can be the cause or consequence of HF, and the directionality of this relationship varies among individual patients and across the spectrum of HF [24]. The ‘ablate and pace’ strategy can correct the rapid and irregular ventricular rate induced by AF, which is beneficial for maintaining cardiac output and reducing cardiac preload. However, AF patients often present with multiple coexisting HF risk factors, such as underlying cardiac diseases, cardiac remodeling, renal insufficiency, and infections [25]. These factors collectively contribute to the refractoriness of HF in AF and may concurrently attenuate the therapeutic efficacy of the ‘ablate and pace’ approach. Our study found that patients with unfavorable efficacy often presented with worse renal function, larger left atrial diameter, and a higher proportion of males, suggesting that the HF in these individuals may not be primarily driven by the abnormal heart rate and rhythm of AF, and thus the benefits of ‘ablate and pace’ therapy may be limited.

Renal function impairment and left atrial enlargement have been demonstrated to not only affect the efficacy of catheter ablation but also predict the risk of HF in patients with AF [26,27]. Pandey et al. confirmed that for every 10 mg/dL increase in creatinine clearance, the risk of heart failure in AF patients decreased by 50% [28]. Hamatani et al. indicated that larger LAD was independently associated with a higher HFH risk, with those having an LAD ≥ 50 mm facing a 2.36-fold higher risk compared to those with a diameter < 40 mm [29]. In this study, 74% of patients with HFH had a baseline eGFR ≤ 60 mL/min/1.73 m^2^, whereas the ratio was only 32% in non-HFH patients. Relative to eGFR > 60 mL/min/1.73 m^2^, an eGFR ≤ 60 mL/min/1.73 m^2^ conferred a 7.9-fold higher risk of HFH after combined therapy. Similarly, an LAD ≥ 50 mm was associated with a 4.8-fold increased risk of HFH events. Interestingly, while previous studies have identified female sex as a significant predictor of HFH and all-cause mortality in AF patients [30,31], this study found that male sex served as an independent predictor of HFH after ‘ablate and pace’ therapy.

The HFH risk-prediction model improved by each added modality. Patients with one or no risk factors from the final model had a relatively favorable outcome following ‘ablate and pace’ therapy, illustrated by a lower incidence of HFH during follow-up. In contrast, the presence of two or more risk factors was associated with significantly worse prognosis. The poorest outcomes were observed in patients with all three risk factors, all of whom experienced HFH within 2 years. These findings suggest that patients likely to derive either favorable or poor therapeutic benefits from “ablate and pace” therapy may be identified. Specifically, patients with more than one risk factor may derive limited benefit from this intervention, though this observation warrants validation in an independent prospective cohort.

## 5. Limitations

This study had several limitations. First, as a single-center, retrospective study with low sample size and limited endpoint number, this work may introduce the risk of overfitting in the multivariate Cox regression analysis, warranting further internal or external validation with larger sample sizes in the future. Second, the follow-up duration was relatively short, potentially leading to an underestimation of long-term safety outcomes, such as lead-related complications. Third, the majority of patients enrolled were over 70 years old; thus, the efficacy of ‘ablate and pace’ therapy in a younger population remains unclear. Moreover, as the study population is predominantly HFpEF/HFmrEF, further investigation with an enlarged HFrEF subgroup sample is required to validate the robustness of our findings. Despite these limitations, this study represents the first systematic analysis of risk factors influencing the efficacy of ‘ablate and pace’ therapy. The developed risk-prediction model holds potential for aiding in the identification of patients with refractory AF and HF who may less likely benefit from this treatment approach.

## 6. Conclusions

Not all patients with refractory AF and HF derive benefits from ‘ablate and pace’ therapy. LAD ≥ 50 mm, eGFR ≤ 60 mL/min/1.73 m^2^, and male sex were likely predictors of poor therapeutic outcomes. Patients without any of these risk factors had minimal HFH risk, whereas ≥2 risk factors increased the risk significantly. These findings may be useful for identifying patients who are less likely to benefit from this combined approach, though prospective studies are needed to validate these results prior to clinical application.

## Figures and Tables

**Table 1:** Clinical characteristics at inclusion of 98 patients receiving LBBAP and AVN ablation, and comparison of patients with and without HFH during follow-up.

Variables	Total (*n* = 98)	HFH (*n* = 19)	No HFH (*n* = 79)	*p*-Value
Age (y)	73.1 ± 8.9	73.6 ± 7.2	73.0 ± 9.3	0.796
Male (%)	41 (41.8)	12 (63.2)	29 (36.7)	0.036
Body mass index (kg/m^2^)	25.9 ± 4.4	25.8 ± 5.7	25.9 ± 4.0	0.885
Systolic blood pressure (mmHg)	129.1± 19.0	129.6 ± 20.8	128.9 ± 18.7	0.889
Average heart rate (beats/min)	93.6 ± 18.7	95.9 ± 15.2	93.1 ± 19.5	0.550
Baseline QRS morphology				
Normal QRS	89 (90.8)	16 (84.2)	73 (92.4)	0.267
RBBB	7 (7.1)	3 (15.8)	4 (5.1)	0.130
LBBB	2 (2.0)	0 (0)	2 (2.5)	>0.999
Hypertension (%)	67 (68.4)	12 (63.2)	55 (69.6)	0.587
Diabetes mellitus (%)	25 (25.5)	8 (42.1)	17 (21.5)	0.065
Coronary artery disease (%)	26 (26.5)	5 (26.3)	21 (26.6)	0.981
eGFR (mL/min/1.73 m^2^)	68.6 ± 21.7	50.6 ± 18.1	73.0 ± 20.3	<0.001
eGFR ≤ 60 mL/min/1.73 m^2^ (%)	39 (39.8)	14 (73.7)	25 (31.6)	0.001
lgNT-proBNP	2.6 ± 0.6	2.7 ± 0.6	2.6 ± 0.5	0.172
CHA_2_DS_2_-VASc score	4.9 ± 1.8	5.0 ± 1.6	4.8 ± 1.9	0.705
HAS-BLED score	2.5 ± 1.1	2.8 ± 1.0	2.4 ± 1.1	0.201
Echocardiogram				
LAD (mm)	47.8 ± 7.2	52.9 ± 9.5	46.6 ± 6.0	<0.001
LAD ≥ 50 mm (%)	30 (30.6)	10 (52.6)	20 (25.3)	0.020
RA dilation (%)	42 (42.9)	13 (68.4)	29 (36.7)	0.012
LVEDd (mm)	48.2 ± 6.5	49.8 ± 7.8	47.8 ± 6.2	0.232
LVEF (%)	54.5 ± 8.0	53.7 ± 8.8	54.7 ± 7.8	0.644
HF phenotypes				
HFpEF	63 (64.3)	12 (63.2)	51 (64.6)	0.909
HFmrEF	24 (24.5)	4 (21.0)	20 (25.3)	0.698
HFrEF	11 (11.2)	3 (15.8)	8 (10.1)	0.483
NYHA class				0.173
II	35 (35.7)	6 (31.6)	29 (36.7)	
III	50 (51.0)	8 (42.1)	42 (53.2)	
IV	13 (13.3)	5 (26.3)	8 (10.1)	
Medications				
β-blocker	83 (84.7)	15 (78.9)	67 (86.1)	0.535
ACEi/ARB/ARNI	64 (65.3)	11 (57.9)	53 (67.1)	0.450
MRA	48 (49.0)	9 (47.3)	39 (49.4)	0.876
Diuretics	51 (52.0)	10 (52.6)	41 (51.9)	0.954
Statin	64 (65.3)	12 (63.2)	52 (65.8)	0.827

Values are given as mean ± SD or *n* (%) unless otherwise indicated.

**Table 2:** Pacing and ablation parameters during the procedure, and comparison of patients with and without HFH during follow-up.

	Total (*n* = 98)	HFH (*n* = 19)	No HFH (*n* = 79)	*p*-Value
Intrinsic QRS duration, ms	97.5 ± 18.4	103.6 ± 22.2	96.1 ± 17.2	0.110
LBBAP QRS, ms	126.1 ± 10.5	126.1 ± 9.2	126.2 ± 10.9	0.986
Stim-LVAT, ms	76.3 ± 9.0	76.8 ± 8.4	76.2 ± 9.2	0.266
Pacemaker				
Single chamber	13 (13.3)	3 (15.8)	10 (12.7)	
Dual chamber	85 (86.7)	16 (84.2)	69 (87.3)	
Total procedural time, min	115.1 ± 26.6	118.9± 27.4	114.2 ± 26.5	0.489
Fluoroscopy time, min	51.9 ± 17.6	54.3 ± 18.3	51.4 ± 17.5	0.527
AVN ablation time, min	4.9 ± 2.0	5.0 ± 1.7	4.9 ± 2.1	0.876
**Baseline pacing parameters**				
Mean impedance, Ohms	761.8 ± 188.5	723.6 ± 138.4	771.2 ± 198.6	0.326
Mean sensed R wave, mV	12.1 ± 3.8	11.0 ± 3.4	12.3 ± 3.9	0.196
Mean threshold, V at 0.4 ms	0.68 ± 0.20	0.63 ± 0.13	0.69 ± 0.21	0.208
Distribution of threshold, V				0.598
<0.5	2 (2.0)	0 (0)	2 (2.5)	
0.5, <1	93 (94.9)	19 (100.0)	74 (93.7)	
1, <1.5	3 (3.1)	0 (0)	3 (3.8)	
≥1.5	0 (0)	0 (0)	0 (0)	

Values are given as mean ± SD or *n* (%) unless otherwise indicated. AVN = atrioventricular node; HFH = hospitalization for heart failure; LBBAP = left bundle branch area pacing; Stim-LVAT = stimulus-to-peak LV activation time.

**Table 3:** Predictors of HFH after LBBAP and AVN ablation in patients with atrial fibrillation and heart failure.

	Univariate Analysis		Multivariate Analysis	
	HR (95%CI)	*p*-Value	HR (95%CI)	*p*-Value
Age, per y	1.0 (1.0–1.1)	0.766		
Male	2.7 (1.1–7.0)	0.034	3.9 (1.5–10.3)	0.006
Hypertension	0.8 (0.3–2.1)	0.679		
Diabetes mellitus	2.3 (0.9–5.7)	0.176		
Coronary artery disease	1.1 (0.4–2.9)	0.931		
Stroke	1.8 (0.7–4.3)	0.223		
eGFR ≤ 60 mL/min/1.73 m^2^	5.0 (1.8–13.8)	0.002	7.9 (2.7–22.9)	< 0.001
lgNT-proBNP	1.7 (0.8–3.6)	0.194		
NYHA function classe	1.5 (0.8–3.0)	0.217		
CHA_2_DS_2_-VASc score	1.1 (0.8–1.3)	0.690		
HAS-BLED score	1.3 (0.9–2.1)	0.196		
LAD ≥ 50 mm	3.0 (1.2–7.3)	0.019	4.8 (1.9–12.5)	0.001
RA dilation	3.3 (1.3–8.8)	0.015	1.8 (0.6–4.9)	0.284
LVEDd, per mm	1.04 (0.98–1.12)	0.172		
LVEF, per %	0.98 (0.93–1.04)	0.553		
Intrinsic QRS duration, per ms	1.02 (0.99–1.04)	0.158		
LBBAP QRS, per ms	1.00 (0.96–1.05)	0.941		
Stim-LVAT, per ms	1.01 (0.96–1.06)	0.738		
β-blocker	0.7 (0.2–2.1)	0.541		
ACEi/ARB/ARNI	0.7 (0.3–1.8)	0.448		
MRA	1.0 (0.4–2.4)	0.920		
Diuretics	1.1 (0.4–2.7)	0.865		
Statin	0.9 (0.4–2.3)	0.825		

AVN = atrioventricular node; eGFR = estimated glomerular filtration rate (calculated by CKD-EPI formula); HFH = hospitalization for heart failure; LAD = left atrial anterior–posterior diameter; LBBAP = left bundle branch area pacing; LVEDd = left ventricular end-diastolic diameter; LVEF = left ventricular ejection fraction; NT-proBNP = N-terminal-pro hormone B-type natriuretic peptide; NYHA = New York Heart Association; RA = right atrium; Stim-LVAT = stimulus-to-peak LV activation time.

## Data Availability

The data presented in this study are available upon request from the corresponding author and are not publicly available due to ethical issues.

## Supplementary Materials

The following supporting information can be downloaded at https://www.mdpi.com/article/10.3390/jcdd13070330/s1. Figure S1: Case example of LBBAP and AVN ablation in a patient with atrial fibrillation with heart failure.
