# Supplementary material for: Predictors of Unfavorable Therapeutic Response to Left Bundle Branch Area Pacing and Atrioventricular Node Ablation in Patients with Atrial Fibrillation and Heart Failure
Source: J Cardiovasc Dev Dis. 2026 Jul 14;13(7):330. doi: 10.3390/jcdd13070330 (PMC13411699; doi:10.3390/jcdd13070330)

Supplementary Material

Lishui Shen, et al. Predictors of therapeutic response to left bundle branch area pacing and atrioventricular node ablation in patients with atrial fibrillation and heart failure.

Figure S1. Case example of LBBAP and AVN ablation in a patient with atrial fibrillation with heart failure. A, Native ECG. **B**, Left bundle branch potential recorded from the 3830 lead (red arrow). **C**, The 3830 lead implanted in LBB successfully; unipolar LBBP at 1 V/0.4 ms resulted in LBB capture with Stim-LVAT of 74 ms which was equal to the intrinsic LBB potential-LVAT (74ms). **D**, Electrogram after AVN ablation. **E**, LBBAP threshold remained unchanged after ablation. Radiograph before (**F**) and 1 years after (**G**) LBBAP and AVN ablation. AVN = atrioventricular node; LBBAP = left bundle branch area pacing.

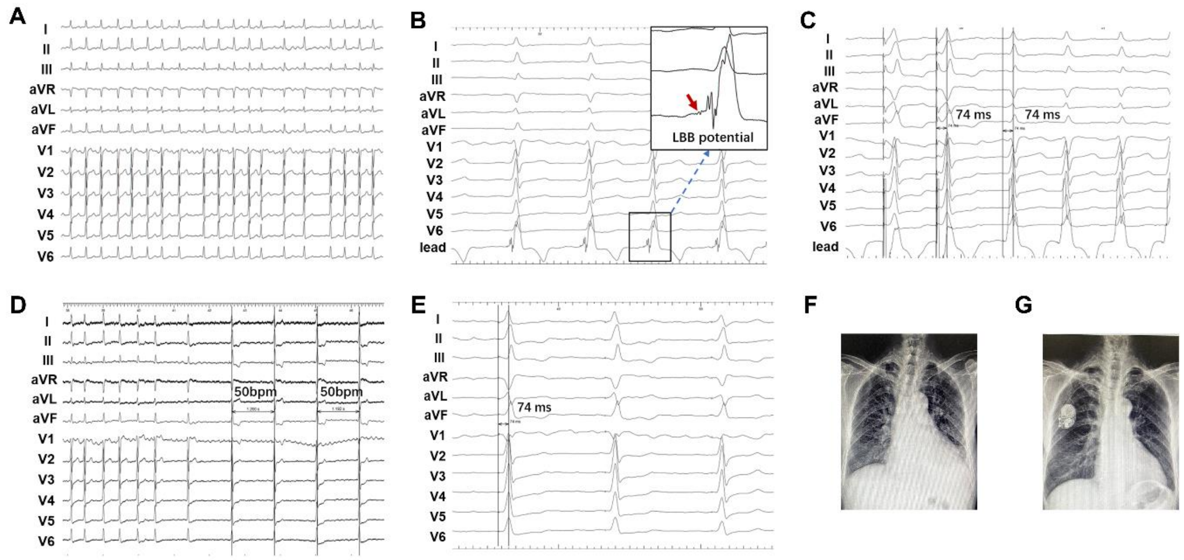

Supplement: Supplementary file 1 [file jcdd-13-00330-s001.zip › jcdd-4270252-supplementary.pdf]
